# Study on Residual Stress and Optimization of Welding Parameters in Linear Friction Welding of TC17 Titanium Alloy

**DOI:** 10.3390/ma15248963

**Published:** 2022-12-15

**Authors:** Hai Gong, Meiqin Liu, Tao Zhang, Yongbiao He, Yunxin Wu, Zhouxiang Yu

**Affiliations:** 1 Light Alloy Research Institute, Central South University, Changsha 410083, China; 2 State Key Laboratory of High Performance Complex Manufacturing, Central South University, Changsha 410083, China; 3 Neutron Scattering Laboratory, China Institute of Atomic Energy, Beijing 102413, China

**Keywords:** TC17 titanium alloy, linear friction welding, temperature field, residual stress, optimized parameters

## Abstract

Linear friction welding (LFW) is a kind of advanced manufacturing technology and used mainly in the manufacturing of aircraft engine bladed disks (blisks) currently. However, the residual stress evolution of TC17 titanium alloy during LFW is complex and its distribution is difficult to characterize. In this study, the residual stress of welding was studied using numerical simulation and experimental methods. The results showed that the maximum temperature on the welded surface was up to 1000 °C and the cooling rates in the lengthwise, widthwise, and normal direction with the same distance from the center of the weld were 456 °C/s, 448 °C/s, and 232 °C/s, respectively. The lengthwise stress on the welding surface was the largest, followed by the widthwise stress and normal stress. Among the three factors affecting welding stress, the upsetting force played a leading role, followed by the vibration amplitude and frequency of the welded parts. By optimizing the process parameters: upsetting force 18.2 kN, vibration amplitude 2.5 mm, vibration frequency 40 Hz, a 30% decrease of the maximum residual stress could be achieved compared to that without optimization. The residual stress before and after welding parameter optimization was measured by the contour method, and the measured results were in good agreement with the simulation results, which verified the effectiveness of parameter optimization on residual stress controlling.

## 1. Introduction

Titanium alloy with high strength, hardenability, corrosion resistance, creep resistance, and fracture toughness is widely used in the aerospace field [1]. Among them, TC17 titanium alloy is a type α+β titanium alloy rich in β phase, with the highest working temperature of 427 °C and a phase transformation temperature of 890 °C. LFW can not only meet the needs of machining, but also meet the needs of repairing [2,3]. The welded joint has high performance: defects such as microcracks and pores are avoided, and the microhardness is higher than that of the base metal [4]. However, in the LFW process, heat and stress always act on the friction interface and the surrounding metals, resulting in various thermal coupling phenomena such as mutual diffusion, dynamic recrystallization, plastic deformation, and flow in the welded joint. This phenomenon easily causes stress concentration in the weld area, and the friction interface and the surrounding of the weld become the weak links of the welded joint due to the high stress level [5]. The residual stress introduced by LFW in the manufacturing of aviation key parts cannot be ignored [6]. The beneficial residual stress is mainly the surface compressive stress, while the influence of residual stress is usually harmful [7,8]. According to its influence on components, it can be roughly divided into the influence on material fatigue, material strength, and other mechanical properties and the influence on dimensional deviation and deformation after material processing. It often leads to the failure of materials, the destruction of components, the collapse of structures, the shortening of service life, etc.

In the early research of LFW, simplified models were often used, ignoring that the interaction between weldments is only represented by adding heat. Although this simplified heating model improves the computational efficiency, it cannot explain some key aspects of thermo-mechanical interaction [9,10,11,12]. Clément Bühr [13] et al. developed the LFW model of TC4 titanium alloy with weld heat applied at the interface, while ignoring the material deformation and discharge captured, by sequentially removing a row of elements. The LFW finite element model of R. Turner [14] et al. considered the elastic-viscoplastic material formula, studied the evolution of residual stress during welding and cooling, and compared it with the measurement results of the X-ray diffraction method. Zhao Pengkang [15] studied the influence of processing parameters on the temperature field and axial shortening of TC11 and TC17 titanium alloy linear friction welded joints by numerical simulation and experimental methods. J. Romero [16] made a detailed study of the development of residual stress of TC4 titanium alloy under different forging forces of LFW.

At present, a great deal of research has been done on LFW numerical simulation and experimentation, but it mainly focuses on the temperature field, deformation, and microstructure after welding. The mechanism of residual stress after welding and the influence of various welding parameters on stress are less and not deep. Based on the research done by others, in order to improve the calculation efficiency and reveal the welding process, this work combines mechanical vibration heat generation with symmetrical surface cooling to study the residual stress after welding.

In this work, the ABAQUS explicit algorithm was used to establish a three-dimensional thermo-mechanical coupling finite element model of LFW. The information of temperature field and stress field was obtained by friction heat generation and symmetrical plane cooling, and the influence of temperature on residual stress, the magnitude, and the distribution of residual stress after welding were revealed. The optimization of the upsetting force, amplitude, and vibration frequency was conducted with the aim of achieving the minimum residual stress of LFW, which provides theoretical guidance for optimizing process parameters. At the same time, the contour method was used to verify the correctness of the model. Based on the three-coordinate measuring instrument, the cutting surface perpendicular to the welding surface was measured, and the deformation surface was reversely applied to the ideal cutting surface to obtain the stress distribution perpendicular to the cutting surface.

## 2. Materials and Methods

### 2.1. Materials

**Figure 1:**
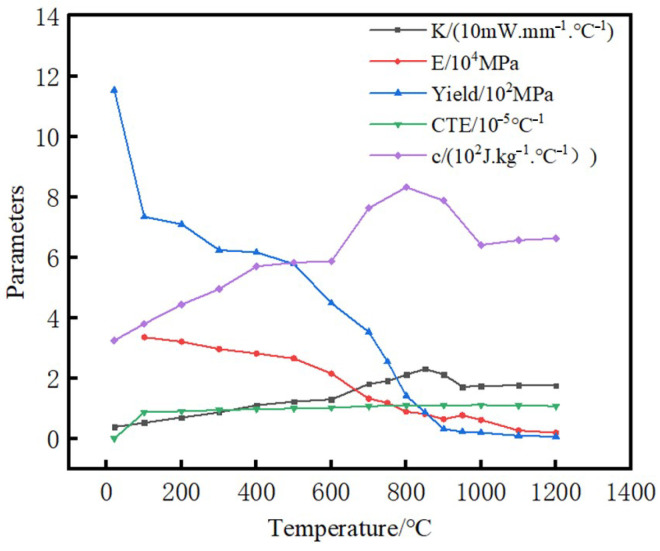
TC17 titanium alloy material parameters.

The material used in the model and experiment was TC17 titanium alloy (Ti-5Al-2Sn-2Zr-4Mo-4Cr). Material parameters are shown in Figure 1, including density (4.68 × 10^−9^ t/mm^3^), Poisson’s ratio (0.34) [17], thermal conductivity K, elastic modulus E, yield strength σ, thermal expansion coefficient CTE, and specific heat c, which are very important for welding stress simulation.

The temperature point of the parameter experiment was from normal temperature to 1200 °C with an interval of 100 °C. IET-1600VP was used to measure elastic modulus E, which was manufactured by the Luoyang zhuosheng detection instrument Co., Ltd, Luoyang, China. The measurement sample size was 120 mm × 45 mm × 12 mm. The yield strength *σ* was measured by isothermal uniaxial compression in a Gleeble 3500 thermal simulator manufactured by the DATA SCIENCES INTERNATIONAL, INC (DSI), MN, USA, with a constant strain rate, heating rate of 10 °C/s, holding time of 3 min, deformation degree of 50%, and true strain of about 0.7. The measurement sample size of thermal expansion coefficient was Φ 5 mm × 25 mm. The measurement sample size of the specific heat capacity and thermal diffusivity was Φ 12.5 mm × 2.5 mm, which was measured by comparison method and flash method, respectively. Combining the specific heat capacity, thermal diffusivity, and density of the material, the thermal conductivity of the material was calculated according to Formula (1)

(1) $$\lambda =\alpha C_{P}\rho$$

where *α* is the thermal diffusivity, *C_P_* is the specific heat capacity, and *ρ* is density.

### 2.2. Methods

#### 2.2.1. Finite Element Model

During the LFW process, one specimen reciprocated with a certain frequency (f) and amplitude (A) under the action of the exciting force, while the other gradually moved to the reciprocating specimen under the action of the upsetting force (F). The pressurized oscillatory motion of the block produces frictional heat and results in the plasticization of the interface material. The plasticized material along with the interface contaminants, such as oxides, is then expelled in the form of flash toward the edges [18,19]. After rubbing the specimens for 3–4 s, the vibration was stopped. The upsetting force was kept for tens of seconds and then unloaded. The specimens on both sides were welded together due to mutual diffusion and recrystallization.

The finite element model of TC17 titanium alloy LFW was established, and the thermo-mechanical coupling process of friction welding can be characterized by three aspects: welding thermal process, plastic rheological field, and stress–strain field. The relationship between material flow stress and temperature, strain and strain rate can be expressed as follows:

(2) $$\sigma =(A+B\varepsilon_{p}^{n})(1+C\ln{\dot{\varepsilon }}^{*})(1-T{*}^{m})]$$

where A, *B*, *n*, *C*, *m* are material constants, $\varepsilon_{P}$ is the effective plastic strain, and ${\dot{\varepsilon }}^{*}$ is the effective plastic strain rate normalized with respect to a reference strain rate (${\dot{\varepsilon }}^{*}=\dot{\varepsilon }/{\dot{\varepsilon }}_{r}$). T* is a homologous temperature defined as $T*=\left(T-T_{r}\right)/\left(T_{m}-T_{r}\right)$, where *T_m_* is the melting point and *T_r_* the reference or transition temperature. The rheological field is mainly used to study axial shortening and deformation, while the residual stress is mainly studied from welding thermal process and stress–strain field. In addition, the rheological field is ignored due to the huge computational workload required in the material extrusion stage.

**Figure 2:**
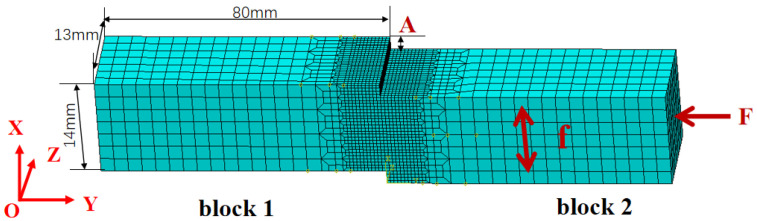
Schematic diagram of LFW.

In LFW simulation, the dimensions of the two blocks for LFW connection were: X × Y × Z = 14 mm × 40 mm × 13 mm, as shown in Figure 2. In which X, Y, and Z are lengthwise, normal directions, and widthwise, respectively. Block 1 was fixed by the full restraint of the left XOZ surface, the upsetting force F (14.56 kN) was applied to block 1 through block 2. At the same time, block 2 (two degrees of freedom with X and Y directions) reciprocated linearly along the X direction with frequency f (40 Hz) and amplitude A (2 mm). Under the action of the upsetting force, the two blocks rubbed against each other for 4 s, then the friction stopped, and finally the force was released, and the welded test block was cooled to room temperature.

C3D8RT hexahedral element was adopted in the finite element model, and the element size away from the weld surface was 2 mm × 2 mm × 2 mm, a smaller size of 0.5 mm × 0.5 mm × 0.5 mm near the weld area was used to obtain an accurate temperature field and stress field. The material parameters for modeling were as shown in Figure 1, and the ratio of interfacial frictional heat to internal energy of workpiece was set to 0.9 [20].

**Figure 3:**
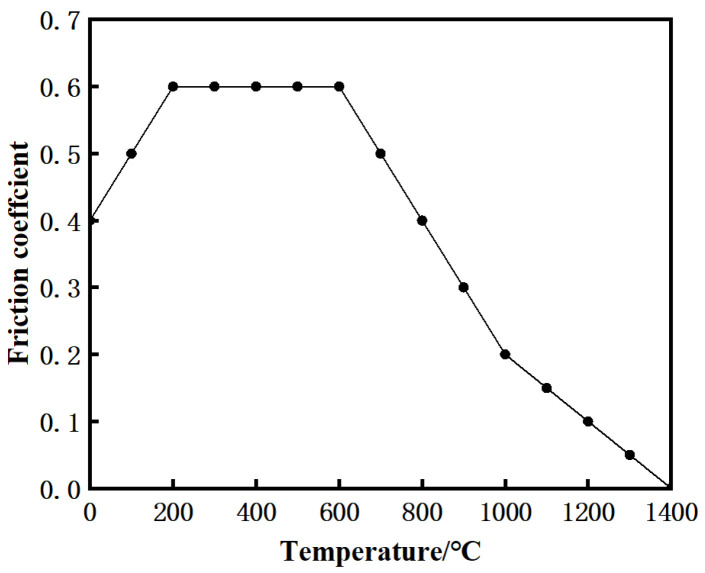
Friction coefficient under different temperatures.

In the friction heat generation stage, the middle welding surface adopts surface-to-surface contact, and the penalty function was selected to describe its friction behavior. The relationship between friction coefficient and temperature is shown in Figure 3 [20]. Since the welded parts were finally cooled in air, the heat exchange coefficient was set to 60 W/(m^2^ °C). In the cooling stage, in order to simulate the welded state and save calculation time, the welding surface of the weldment (because the temperature field and stress field of the two friction surfaces was the same) was set as a symmetrical surface, and the heat exchange with air was set on the other five surfaces except the symmetrical surface.

#### 2.2.2. Experiment

**Figure 4:**
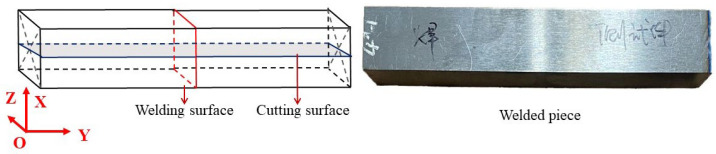
Schematic diagram of welding surface and cutting surface.

**Figure 5:**
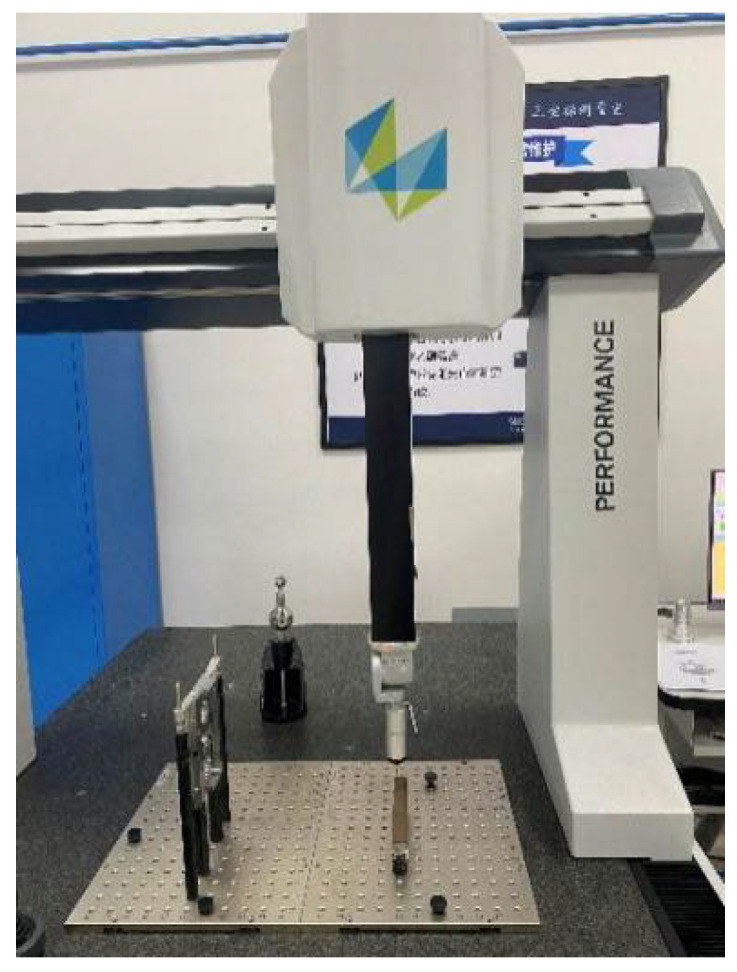
The three-coordinate measuring instrument diagram.

The LFW experiment was conducted on LFW-20T equipment, self-developed by AVIC Manufacturing Technology Institute, Beijing, China. The shape and size of the blocks in the LFW experiment were consistent with the simulation, and welding parameters of F = 14.56 kN, f = 40 Hz, and A = 2 mm were adopted. After welding, the contour method was used to test the distribution of residual stress in the center of the welded part, and the specimen is shown in Figure 4. The welding surface is the friction contact surface of two blocks, and the cutting surface is the YOZ surface perpendicular to the center of the welding surface. In the contour method experiment, the SDKA machine tool produced by Suzhou Sanguang Technology Co. Ltd., Suzhou, China was used to cut the specimen, and the diameter of the molybdenum wire was 0.25 mm. After cutting, the deformation of the cutting surface was measured by a three-coordinate measuring instrument made by the HEXAGON, Qingdao, China with an accuracy of 2 μm/300 mm. The measuring process is shown in Figure 5. The measured displacement of the scattered points of the contour was 2 mm. The measured deformation was reversely loaded perpendicularly to the cutting surface in a finite element model, and the stress distribution perpendicular to the cutting surface (the stress parallel to the lengthwise of the welding surface) was obtained.

## 3. Results and Discussions

### 3.1. Evolution of the Temperature and Its Influence on Stress

**Figure 6:**
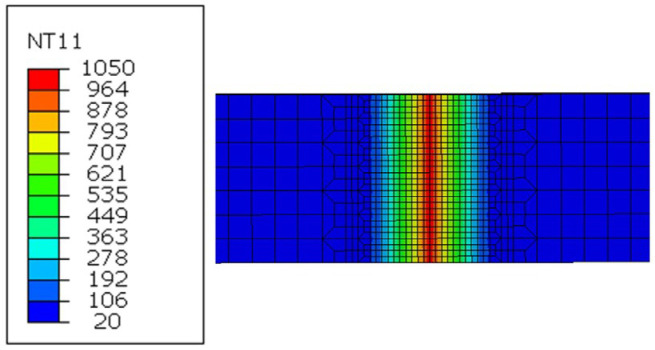
Instantaneous temperature distribution of cutting surface after friction.

Under the above welding parameters, the instantaneous temperature field generated after friction (4 s) is shown in Figure 6, and the weld temperature is above 1000 °C. It can be seen that the temperature distribution of the welding surface is relatively uniform, and the width of the high-temperature zone of the weld is about 1.5~2 mm [16,21,22,23].

**Figure 7:**
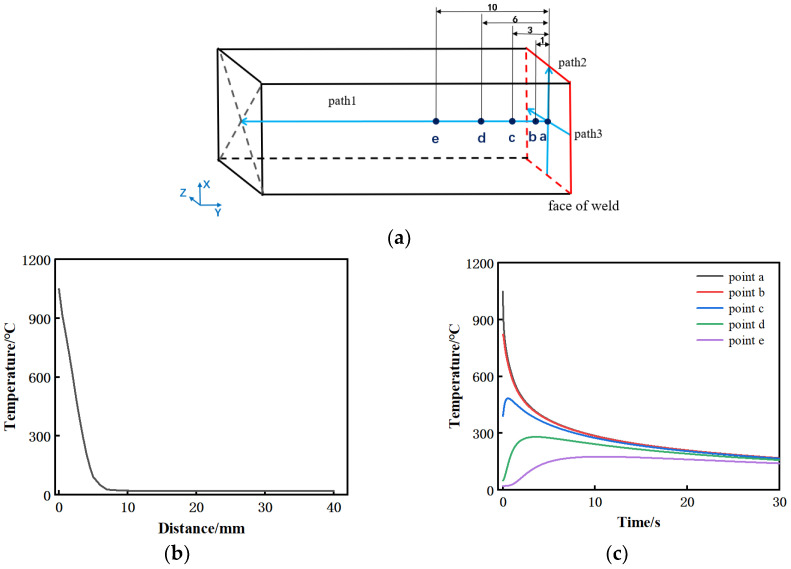
Temperature distribution during the cooling process: (**a**) Schematic diagram of path and key points; (**b**) temperature distribution along path 1 when cooling starts; (**c**) temperature time curve of key points in early cooling period (within 30 s).

Five points a, b, c, d, and e were selected to further analyze the temperature evolution in the workpiece during the cooling process. These five points are respectively 0 mm, 1 mm, 3 mm, 6 mm, and 10 mm away from the weld surface, and they are located on the same path perpendicular to the weld surface, as shown in path 1 in Figure 7a. The results show that the temperature gradient at the welding seam is large, and the temperature at 7 mm from the welding surface is near room temperature. When cooling, the highest temperatures at point a and b are 1050 °C and 820 °C, respectively. Due to heat transfer, the temperature in this area drops extremely fast, and the temperature from near to far from the welding surface rises in turn. In addition, the temperature in a–b is higher than the phase transition temperature and located in the center of the weld, and this area is prone to plastic deformation due to the high temperature, so it is inferred that this area is the weld zone. The peak temperature at c is close to 500 °C, and at this temperature, a tiny deformation is produced by the upsetting force, so it is inferred that this area is the heat affected zone. The whole temperature at d is lower than 300 °C, and the heat transfer effect is small. Therefore, it is inferred that the area further away from the welding surface is the base material area.

**Figure 8:**
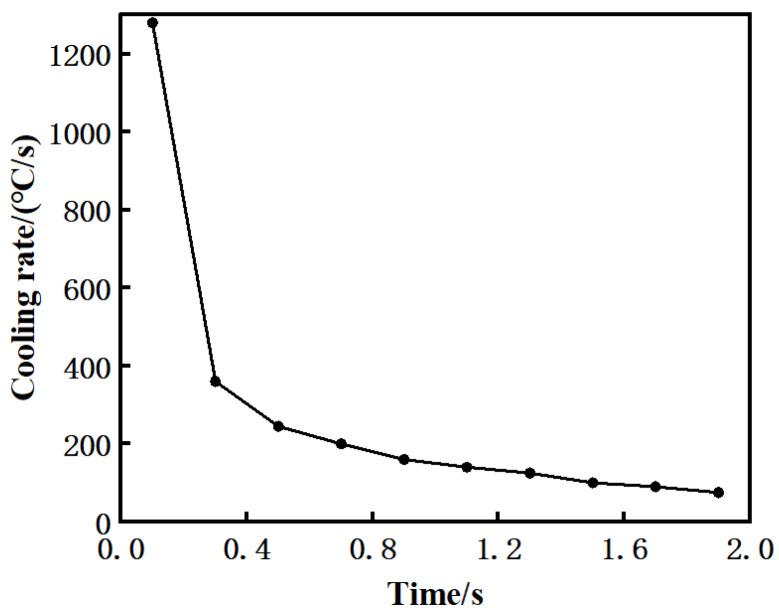
Cooling rate of path 2 with cooling time of 0–2 s.

The temperature field generated on the welded surface after friction is relatively uniform, and the lengthwise and widthwise temperature of the model on the welded surface are similar, so the path in one direction of the lengthwise is selected to study the temperature on the welded surface. The path was established parallel to the lengthwise of the welding surface through the center of the welding surface, as shown in path 2 in Figure 7a. Because the temperatures on the path are almost the same, the cooling rate of the average temperature on the path after cooling for 2 s is calculated, as shown in Figure 8. Before cooling, the cooling rate reaches 1280 °C/s within 0.2 s. After cooling, the cooling rate gradually decreases, and it decreases to 75 °C/s when it is close to 2 s. It can be seen that the cooling rate is extremely high at the early stage of the end of friction, so it is speculated that this stage is the main cause of welding residual stress.

**Figure 9:**
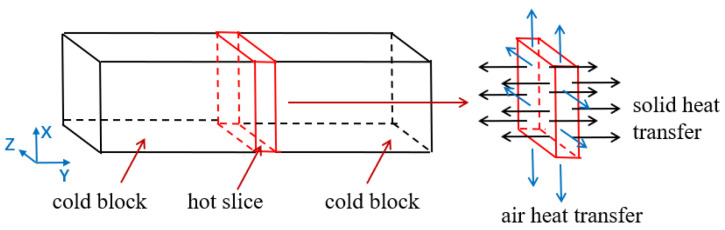
Simplified schematic diagram of cooling.

Heat-induced strain is mainly used to analyze welding residual stress [14], which is due to the difference of the thermal expansion and contraction of materials during heating and cooling, and its main mechanism is the heat-induced strain during cooling after oscillation [24]. During the analysis, the cooling process of the model can be simplified as Figure 9, which is a hot slice of about 2 mm sandwiched between two cold blocks [15]. From Figure 6, it can be seen that the temperature at both ends is not much different from the central temperature, while Figure 7c shows that the temperature gradient perpendicular to the welding surface is large, so it can be inferred that the heat transfer coefficient between the slice and the cold block is far greater than that between the slice and the air. Therefore, the heat is mainly conducted from the left and right directions, along which the temperature has a transition stage. Even though the weld will produce tensile stress in the normal direction, the tensile stress is relatively small due to the “buffering” effect of the cold plate. However, the temperature of the slice along the lengthwise and widthwise of the welding surface is greatly different from that of the air, and the early cooling rate reaches 1280 °C/s, which leads to the extremely rapid temperature drop and the sharp shrinkage of the slice, resulting in a great tensile stress along the two directions of the welding surface. It can be inferred that the tensile stress along the two directions of the welded surface after LFW cooling is much greater than the tensile stress perpendicular to the welded surface. Therefore, the center of the hot slice perpendicular to three directions is selected in the simplified model, and the cooling rates of these three points are derived as the cooling rates in the corresponding directions. According to the above analysis, the average rate of each point within 1 s is selected for calculation (it can be known that the cooling rate in the initial cooling stage is extremely high, which is the main stage of residual stress generation). The cooling rates in the direction parallel to the lengthwise, widthwise, and perpendicular to the welded surface are 456 °C/s, 448 °C/s, and 232 °C/s, respectively, which more intuitively shows that the residual stress is the smallest in the direction perpendicular to the welded surface.

### 3.2. Evolution of the Stress

**Figure 10:**
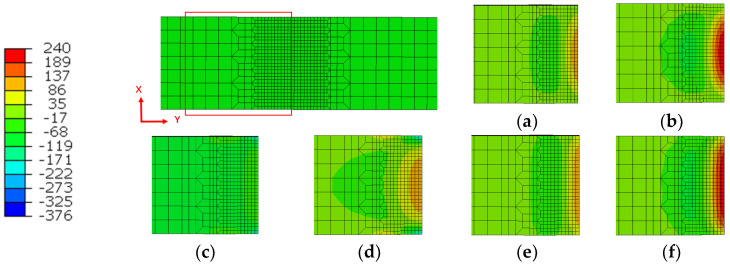
Stress field of cutting surface during the cooling process; (**a**) X stress with cooling time of 1 s; (**b**) X stress after cooling process; (**c**) Y stress with cooling time of 1 s; (**d**) Y stress after cooling process; (**e**) Z stress with cooling time of 1 s; (**f**) Z stress after cooling process.

**Figure 11:**
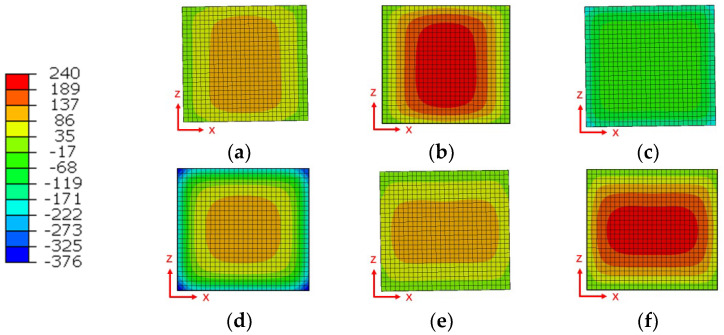
Stress field of welding surface during the cooling process; (**a**) X stress with cooling time of 1 s; (**b**) X stress after cooling process; (**c**) Y stress with cooling time of 1 s; (**d**) Y stress after cooling process; (**e**) Z stress with cooling time of 1 s; (**f**) Z stress after cooling process.

After the end of friction, the stress distribution in three directions along the two surfaces of the model (Figure 4) at the time of cooling for 1 s and at the end of cooling are shown in Figure 10 and Figure 11, respectively. The results show that after cooling for 1 s, the stress in two directions of the weld surface reaches 118 MPa, which is half of the final residual stress, while the vertical welding surface keeps a certain compressive stress almost unchanged due to the influence of upsetting force. After cooling, the stresses in three directions are concentrated in the weld zone, in which the maximum residual stress in two directions of the welded surface is about 240 MPa, and the maximum tensile stress in the vertical welded surface is 110 MPa, which is smaller than the other two directions.

**Figure 12:**
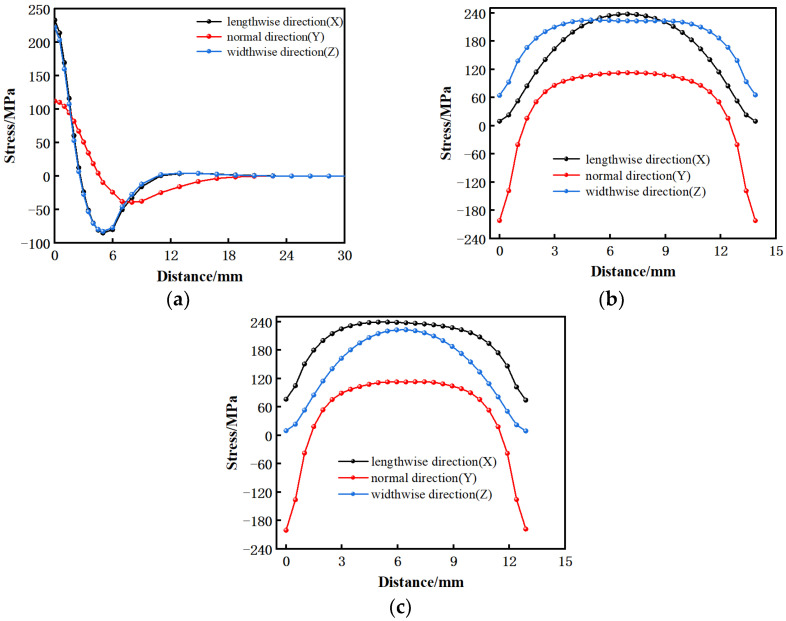
The residual stress distribution after cooling process; (**a**) path 1; (**b**) path 2; (**c**) path 3.

To further understand the magnitude and change of the residual stress, a path was established along the vertical welding surface from the weld center, and two paths were built parallel to both sides at the center of the over-welded surface, which were named paths 1, 2, and 3, in turn, as shown in Figure 7a, and the stresses of the three paths in three directions were derived as shown in Figure 12. Figure 12a shows that the trend and distribution of residual stress in this work are consistent with Turner, R., Romero, J., Frankel, P. and others [14,16,25,26,27]. For example, in Turner, R.’s work, the tensile stress is near the weld and the maximum tensile stress is at the center of the weld. The compressive stress is at 2–6 mm away from the weld, and the stress is zero at a certain distance from the weld and further away. Because the sample size is larger on the long side of the parallel welding surface, the test result in this direction is close to twice that of this work. At the same time, the residual stress along the upsetting force direction is the smallest. In this work, there is a small difference in stress in two directions along the welding surface, with the maximum tensile stress 240 MPa in the center of the weld. This is due to the tensile stress caused by material shrinkage when the weld temperature is too high. The compressive stress at 3–10 mm is to balance the tensile stress at the weld. The position far away from the weld is less affected by temperature, so it is in a stress-free state. However, the stress in the direction perpendicular to the welding surface is the smallest of the three directions, and the maximum tensile stress is about 110 MPa, which is due to the uniform expansion and contraction of the material in this direction. It can be seen from Figure 12b that the stress along the path parallel to the lengthwise at the center of the over-welded surface is mainly concentrated in the center, and the stress at both ends is small. This is because the temperature difference between the welded seam and the air is great, and the sharp contraction during cooling produces great tensile stress. The center is kept at a high stress level due to the restricted stress at both ends, while the unconstrained stress at both ends is released, especially in the direction of the lengthwise of the welded surface, the stress at both ends is directly and completely released to close to zero. The stress distribution of the path parallel to the widthwise through the center of the welding surface is similar to that of the path parallel to the lengthwise. Generally speaking, the residual stress is the largest in the lengthwise of the welding surface, followed by the widthwise of the welding surface, and the smallest is in the direction perpendicular to the welding surface.

**Figure 13:**
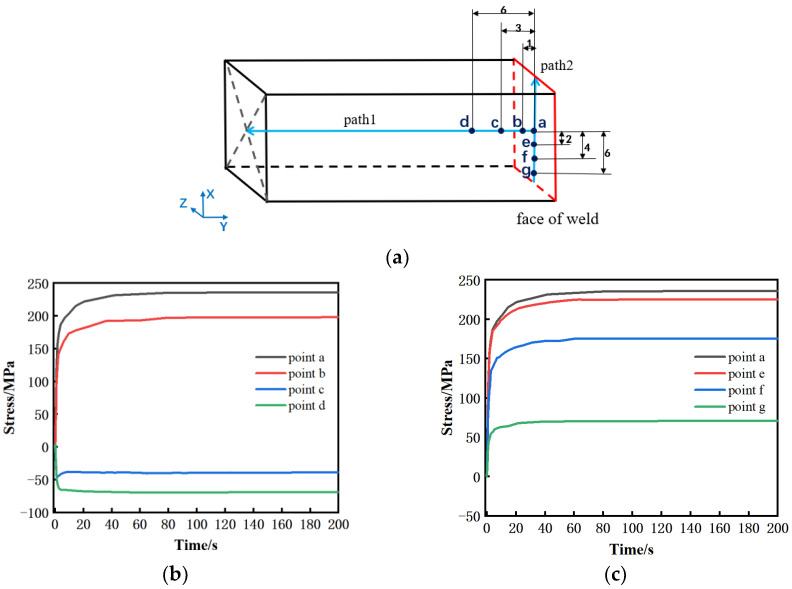
Stress time curve of key points in the lengthwise direction in the early cooling period (within 200 s); (**a**) points diagram; (**b**) key points in path 1; (**c**) key points in path 2.

Take four points at 0 mm, 1 mm, 3 mm, and 6 mm from the welding surface on path 1. Because the stress distribution on path 2 is similar to that on path 3, only three points at 2 mm, 4 mm, and 6 mm from the weld center on path 2 are taken. Extract the stress change curves of these points along the lengthwise of the welding surface, as shown in Figure 13. It can be seen from Figure 13b,c that the stress along the lengthwise direction, whether perpendicular to the welded surface or along the welded surface path, increases slowly from the 5th to the 20th second of cooling, and the residual stress is basically stable at the 20th second.

### 3.3. Optimization of Welding Process Parameters and Verification

#### 3.3.1. Optimization of Welding Process Parameters

The three main welding parameters of LFW are the upsetting force, amplitude, and vibration frequency. At present, scholars [14,16,26,28] have studied the influence of the upsetting force on residual stress. For the determination of the three welding parameter ranges, we refer to others’ references [29,30] and the actual welding situation. The welding process is optimized by the orthogonal test method of three factors and three levels. There are three factors: upsetting force, amplitude, and vibration frequency. There are three levels of upsetting force: 10.92 kN, 14.56 kN, and 18.2 kN, three levels of amplitude: 1.5 mm, 2 mm, and 2.5 mm, and three levels of vibration frequency: 35 Hz, 40 Hz and 45 Hz.

**Figure 14:**
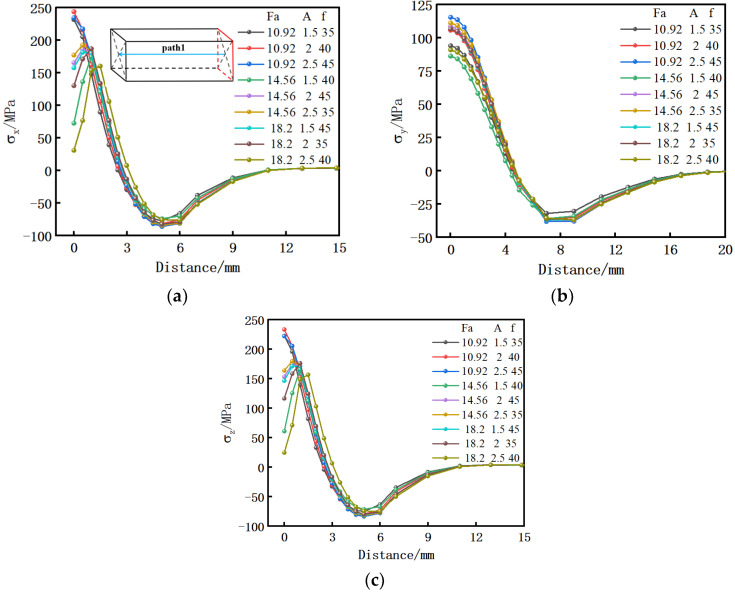
Stress distribution on path 1 of different welding parameters after cooling; (**a**) X direction; (**b**) Y direction; (**c**) Z direction.

In order to optimize the welding parameters and find out how to combine the welding parameters to minimize the residual stress, a three-factor and three-level orthogonal test simulation scheme is adopted. Taking path 1 in Figure 13a as an example, the stress on this path is derived from different parameters, and the stress comparison diagram in three directions is drawn as shown in Figure 14, and the maximum tensile stress of each group of parameters is extracted. The specific parameters are shown in Table 1.

It can be seen from Figure 14 that different upsetting forces, amplitudes, and frequencies have great influence on the residual stress in both sides of the welding surface, but little influence on the direction perpendicular to the welding surface.

After taking the influence of the welding parameters on the final residual stress as the research object, the data in Table 1 are now subjected to a range analysis, as shown in Table 2. When the range method is used for data analysis, if the range R of one column is the largest, it means that when the value of that column changes within the test range, the value change of the corresponding test result is also the largest.

It can be seen from Table 2 that the significant influence of welding parameters in the lengthwise and widthwise of the welding surface on the residual stress after welding is as follows: F>A>f, the significant influence of welding parameters in the direction perpendicular to the welding surface is as follows: f>A>F. Because the residual stress perpendicular to the welding surface is small, and the influence of three welding parameters on the residual stress is small, but the influence of the upsetting force parallel to the welding surface is far greater than that of the other two welding parameters, the upsetting force has the greatest influence on the residual stress in general. It can be seen from Table 2 that when the upsetting force increases, the residual stress in three directions decreases; when the amplitude increases, the residual stress in three directions increases at first and then decreases; when the vibration frequency increases, the residual stress in three directions decreases at first and then increases. Because the residual stress on both sides of the welding surface is far greater than the residual stress in the direction perpendicular to the welding surface, the stress in two directions of the welding surface is mainly considered when analyzing the optimal combination of welding parameters. Therefore, the vertical direction of the welding surface is ignored, and the weights along the lengthwise and the widthwise of the welding surface are 60% and 40%, respectively. After calculation, the optimal combination of the above welding parameters is the upsetting force of 18.2 kN, amplitude of 2.5 mm, and vibration frequency of 40 Hz. Therefore, the final residual stress generated by using the above-mentioned model 9 in the orthogonal test is the smallest. At this time, the maximum residual stress along the lengthwise of the friction surface on path 1 perpendicular to the center of the welding surface is 160 MPa, the maximum residual stress along the widthwise of the friction surface is 157 MPa, and the maximum residual stress perpendicular to the friction surface is 91 MPa. Compared with that before optimization, the residual stress in the two directions parallel to the welding surface decreased by 30%.

#### 3.3.2. Experimental Verification

**Figure 15:**
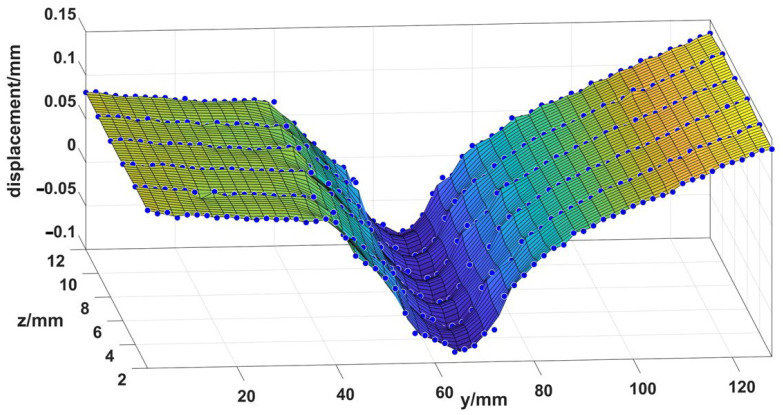
Displacement of the cutting surface under initial welding parameters.

**Figure 16:**
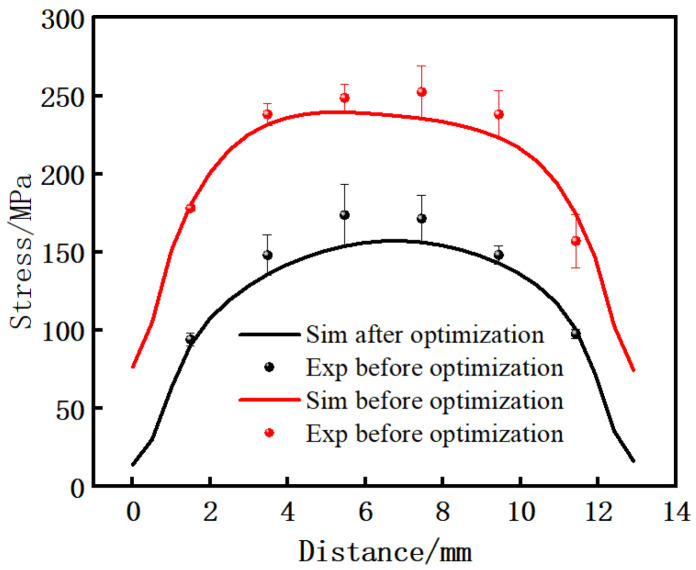
Distribution of the residual stress in the lengthwise direction parallel to the welding surface and 1 mm away from the welding surface under the two welding parameters.

The initial welding parameters (F = 14.56 kN, A = 2 mm, f = 40 Hz) and welding parameters with minimum residual stress (F = 18.2 kN, A = 2.5 mm, f = 40 Hz) were adopted. The measured displacement of scattered points on the contour is shown in Figure 15, with an interval of 2 mm, and the stress field perpendicular to the cutting surface was obtained. The maximum residual stress parallel to the weld seam on the cutting surface measured by experiment was extracted and compared with the maximum residual stress of numerical simulation. As shown in Figure 16, it can be seen that the trend and magnitude of residual stress of experiment and simulation are similar, and the absolute error before and after optimization is about 2–20 MPa. By calculation, the maximum relative error between the simulated value and the experimental value at the center is 11.5%, and the relative error is controlled within 15%, which is in good agreement. From the experimental comparison, the maximum residual stress before optimization is 252 MPa, and the maximum residual stress after optimization is 174 MPa, with a difference of about 78 MPa.

## 4. Conclusions

A finite element model of TC17 titanium alloy with LFW was established. The methods of friction heat generation and symmetry plane cooling were used to simulate the residual stress variation and verified by contour experiment. The following conclusions were drawn:

(1) In the LFW process, the maximum temperature on the welding surface was up to 1000 °C, and the width of high-temperature zone was about 1.5 mm–2 mm.
(2) At the first second of cooling, the stress increased rapidly to 118 MPa, which reached half of the final maximum residual stress of 240 MPa. The stress increased slowly from the 5th to the 20th second of cooling, and the residual stress was basically stable at the 20th second.
(3) The greater residual stress was caused by the faster cooling rate. In the first second of the cooling process, the average cooling rates in the lengthwise, widthwise, and the normal direction of the welding surface were 456 °C/s, 448 °C/s, and 232 °C/s, respectively. Therefore, the residual stress was the largest in the lengthwise direction, followed by the widthwise direction, and the smallest in the normal direction.
(4) The welding parameters affected the magnitude of the residual stresses generated due to LFW. The upsetting force played a dominant role in the variation of residual stress compared to the amplitude and vibration frequency. The maximum residual stress after welding could be reduced by about 70 MPa, which meant a reduction of 30%, when the optimum process parameters with an upsetting force of 18.2 kN, amplitude of 2.5 mm, and vibration frequency of 40 Hz were adopted.

## Figures and Tables

**Table 1:** Maximum residual stress with different welding parameters.

	Factor	F (kN)	A (mm)	f (Hz)	Max $\sigma_{x}$ (MPa)	Max $\sigma_{y}$ (MPa)	Max $\sigma_{z}$ (MPa)
Model							
1		10.92	1.5	35	232.03	94.15	222.74
2		10.92	2	40	244.12	105.84	233.72
3		10.92	2.5	45	234.88	115.66	222.43
4		14.56	1.5	40	175.11	86.36	166.03
5		14.56	2	45	186.39	108.73	175.08
6		14.56	2.5	35	192.90	111.30	180.07
7		18.2	1.5	45	180.59	106.85	171.56
8		18.2	2	35	187.19	106.85	176.37
9		18.2	2.5	40	160.53	91.07	156.95

**Table 2:** Range of residual stress.

	X Direction			Y Direction			Z Direction		
	F	A	f	F	A	f	F	A	f
K1	711.03	587.73	612.12	315.65	287.36	312.3	678.89	562.33	579.18
K2	554.40	617.7	579.76	306.39	321.42	283.27	521.18	585.17	556.70
K3	528.31	588.31	601.86	304.77	318.03	331.24	504.88	559.45	569.07
T1	237.01	195.91	204.04	105.22	95.79	104.1	226.30	186.78	193.06
T2	184.80	205.9	193.25	102.13	107.14	94.42	173.73	195.06	185.57
T3	176.10	196.10	200.62	101.59	106.01	110.41	168.29	186.48	189.69
R	60.91	9.99	10.79	3.63	11.35	15.99	58.01	8.58	7.49

## Data Availability

Not applicable.
